# The Use of Monodisperse Poly(propylene glycol)‑8 as a Polymeric Additive: Effect on the Gelation Temperature and Rheological Properties of Pluronic Hydrogels

**DOI:** 10.1021/acsomega.5c02773

**Published:** 2025-07-28

**Authors:** Zuzanna Samol, Erik Agner, Magne O Sydnes

**Affiliations:** † Department of Chemistry, Bioscience, and Environmental Engineering, 56627 University of Stavanger, Stavanger 4036, Norway; ‡ 478421 Polypure AS, Martin Linges vei 25, Fornebu 1364, Norway; § Department of Chemistry, University of Bergen, Bergen 5020, Norway

## Abstract

Pluronic F127 is widely used for hydrogel preparation, but its low gelation temperature (21 °C at a concentration of 25 wt %) and limited ability to deliver hydrophobic drugs hinder medical applications. A standard approach to address these limitations involves combining Pluronic F127 with other polydisperse polymers, further increasing the system complexity. This study demonstrates the use of monodisperse and high-purity poly­(propylene glycol)-8 (PPG-8), obtained via cost-effective chromatographic purification, as a polymeric modifier. The effect of PPG-8 addition to Pluronic F127, varying from 5 to 20 parts (w/w), was assessed via the vial tilt method and oscillatory rheology. The incorporation of PPG-8 increased the gelation temperature from 21 to 31 °C. The impact of PPG-8 addition on the release of small hydrophilic and hydrophobic molecules was also studied. In the presence of PPG-8, the cumulative release of a hydrophobic small molecule increased from 20% to 60%. Contrastingly, the initial burst release of a small hydrophilic molecule was reduced from 81% to 56% in the first 10 min. These findings showcase the use of high-purity modifiers such as PPG-8 to fine-tune the properties of Pluronic hydrogels, enabling more reproducible formulations for potential clinical use.

## Graphical abstract

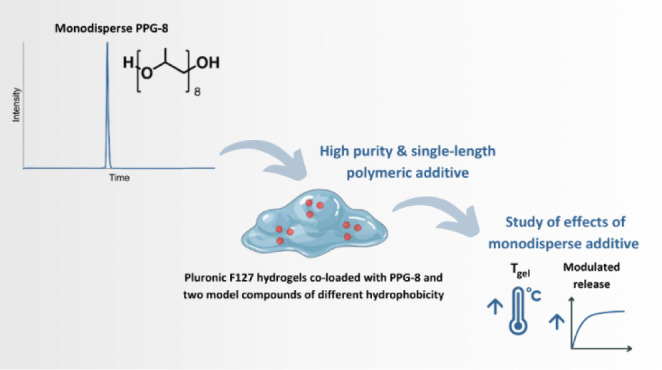

## 1 Introduction

Pluronic F127 is widely employed in the medical field due to its thermoresponsive properties, biocompatibility, and low toxicity.^1,2^ Pluronic F127 consists of approximately 100 units of poly­(ethylene glycol) (PEG) and 65 units of poly­(propylene glycol) (PPG), with an average molecular weight of 12600 g/mol.^1,3^ However, it still possesses some limitations, such as a low gelation temperature (*T*_gel_). For aqueous solutions of Pluronic F127 at a concentration of 25 wt %, the *T*_gel_ is around 21 °C.^1,4^ The ideal formulation should remain liquid below 25 °C for easy application and form a gel under physiological conditions, either at skin or body temperature.^5–7^ Another downside is its limited capacity for loading and releasing drugs, particularly hydrophobic molecules.^8^

These limitations can be overcome via chemical and physical methods.^6,9^ For instance, Pluronics can be modified by covalent binding with other polymers^10^ or by incorporating drug-loaded nanoparticles,^11^ this may lead to safety and toxicity concerns due to the generation of a new complex material. Physical incorporation of other polymers might be a safer alternative.^6^ Common approaches include combining Pluronics of different classes^12^ or adding other polymers, like chitosan^13^ or hyaluronic acid.^14^ Modulating the release of lipophilic molecules (e.g., ibuprofen,^2^ rutin,^5^ and curcumin^15^) can also be achieved by the use of cosolvents.^2,5,15,16^ Despite these efforts, the effects are often limited since the amount of polymeric additives used rarely exceeds 5 wt %.^9^ More importantly, the incorporation of high molecular weight and polydisperse polymers increases the complexity of the system, introduces new impurities, and negatively affects quality and reproducibility.

PPG has previously been utilized to modify the properties of Pluronic F127 hydrogels.^17,18^ It is more hydrophobic than PEG because of the repeating methyl groups in the monomer unit. The polar ether chain can aid the solubilization of hydrophobic compounds, which is especially the case for low molecular weight PPG.^19^ Although PPG is not widely used in pharmaceuticals, where propylene glycol is preferred, PPG with MW = 200–2000 g/mol is employed in cosmetics and personal care products as a humectant, skin conditioning agent, and solubilizer.^20,21^ In these applications, it is recognized as safe for use in concentrations up to 50%.^20^ Unlike other modifiers, adding PPG does not result in the inclusion of a new moiety since PPG is already a central block in Pluronics. Malmsten et al.^17^ found that incorporating 10 wt % PPG with MW = 400 g/mol (PPG400) can enhance the stability of Pluronic F127 hydrogels by increasing the melting temperature from 60 °C to almost 80 °C. In another study, it was found that the critical micelle concentration (CMC) decreases from 0.26 to 0.15 wt % with increasing content of PPG400 from 10 to 30 wt %, thereby promoting micelle formation.^18^

Until now, only polydisperse PPG had been employed in studies since it was not available in a monodisperse form. Commercial PPG400 (Figure 1a) is characterized by moderate to high polydispersity, with the presence of unsaturated impurities stemming from the polymerization process.^19^ Recently, we presented monodisperse PPG (Figure 1b) obtained via chromatographic purification.^22^ Herein, single-length PPG-8 is used as a polymeric modifier of Pluronic F127 hydrogels. Additions of PPG-8 ranging from 5 to 20 parts (w/w) were studied at a fixed concentration of 25 parts (w/w) of Pluronic F127. To better reflect clinical use, where hydrophobic drugs are employed, hydrogels containing both PPG-8 and a small hydrophobic molecule, namely nonivamide, were prepared. The formulations were characterized in terms of *T*_gel_, rheological properties, and cumulative release of nonivamide into PBS buffer. The incorporation of PPG-8 resulted in higher *T*_gel_, both alone and in the presence of nonivamide. Rheological studies revealed the maximum possible incorporation of PPG-8 to facilitate gel formation at body temperature. The release of nonivamide, compared with unmodified hydrogels, was also increased. The effect of PPG-8 on modifying the release of coloaded molecules was further studied by employing a small hydrophilic molecule, similar in molecular weight and chemical structure to nonivamide, namely benzyl 1-poly­(ethylene glycol)-4 (bnPEG-4). In the presence of PPG-8, the initial burst release of hydrophilic bnPEG-4 was decreased. This demonstrated that PPG-8 can both enhance and retard the release of incorporated molecules from the hydrogel matrix, depending on the character of the molecules used molecules.

**Figure 1:**
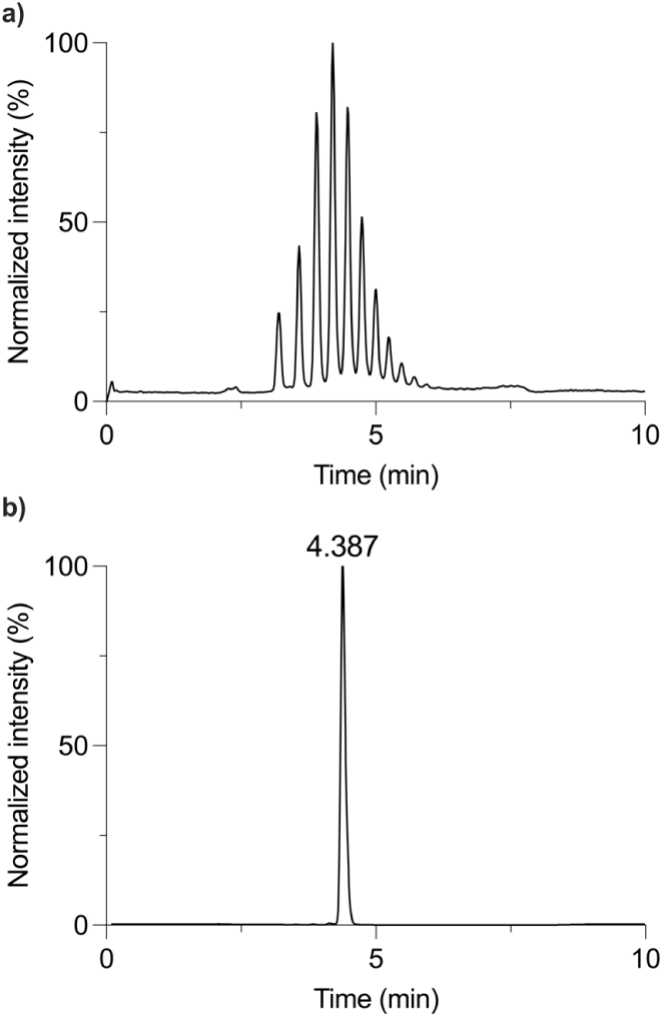
Chromatograms of (a) commercial PPG400 and (b) monodisperse PPG-8.

## 2 Experimental Section

### 2.1 Materials

Pluronic F127 (average MW = 12600 g/mol, Sigma-Aldrich) and nonivamide (MW = 293.4 g/mol, Sigma-Aldrich) were used as received. Benzyl 1-poly­(ethylene glycol)-4 (bnPEG-4, MW = 240.3 g/mol) was provided by Polypure AS. Poly­(propylene glycol)-8 (PPG-8, MW = 482.7 g/mol) was obtained from polydisperse poly­(propylene glycol) (PPG400, average MW = 400 g/mol, Carl-Roth) and purified through sample displacement chromatography, as reported earlier.^22^ PPG400, PPG-8, nonivamide, and bnPEG-4 were characterized by HPLC/MS (Figure S1).

The hydrogel formulations were prepared using Milli-Q water. The release of nonivamide from hydrogels was studied in PBS buffer (Sigma-Aldrich), pH 7.4 using dialysis bags (Spectra/Por 1, MWCO = 6000–8000 g/mol, Spectrum Laboratories Inc.).

### 2.2 Instrumentation

*T*_gel_ measurements were conducted using a Huber Ministat 230 water bath equipped with a PT100 temperature sensor.

The rheological measurements were performed using an Anton Paar MCR 702e MultiDrive rheometer with a PTD 180 MD Peltier temperature control system. All measurements were conducted using a cone-plate geometry (CP50–1/TI, *d* = 49.969 mm, 0.990° angle) at a 0.01 mm gap.

The HPLC/MS analysis was performed using an Agilent 6130 Single Quadrupole LC/MS System with a C18 column (Avantor ACE, 2.1 mm internal diameter, 3 μm particle size). The MS was operated with an electrospray ionization source (ESI) in positive mode, with a drying gas at 350 °C and a flow rate of 12.0 L/min, a capillary voltage of 3000 V, a nebulizer gas pressure of 35 psig, and an acquisition range of 100–3000 *m*/*z*. The mobile phase consisted of Milli-Q water with 0.1% trifluoroacetic acid (solvent A) and acetonitrile (solvent B). For the analysis of nonivamide, the gradient of acetonitrile was adjusted as follows: 0 min, 5% solvent B; 5 min, 75% solvent B; 10 min, 95% solvent B; 12 min, 5% solvent B. For the analysis of bnPEG-4, the gradient of acetonitrile was adjusted as follows: 0 min, 5% solvent B; 5 min, 45% solvent B; 10 min, 95% solvent B; 12 min, 5% solvent B. The analyses were conducted at a flow rate of 0.5 mL/min, with an injection volume of 1 μL, and the column temperature maintained at 25 °C.

### 2.3 Preparation of Pluronic F127 Hydrogel Formulations

The protocol for hydrogel preparation was adapted from Abdeltawab et al.^7^ The respective amounts of Pluronic F127, PPG-8, and nonivamide or bnPEG-4 were measured and dissolved in MilliQ water. The composition of all hydrogels is listed in Table 1. A fixed amount of Pluronic F127 was used, i.e., 25 parts (w/w). A neat hydrogel containing only Pluronic F127 was prepared, resulting in sample F127. The amount of PPG-8 added equaled 5, 10, 15, and 20 parts (w/w), giving samples PPG5, PPG10, PPG15, and PPG20. Additional formulations with 16, 17, 18, and 19 parts (w/w) of PPG-8 were prepared for rheological evaluation, resulting in samples with the following names: PPG16, PPG17, PPG18, and PPG19. The amount of nonivamide and bnPEG-4 was kept constant in all formulations at 0.5 parts (w/w). The hydrogels with nonivamide were prepared by adding 0.01 parts (w/w) of methanol to ensure complete dissolution of the hydrophobic compound.^2,3,23^ Such a small addition did not appear to alter the rheological properties (see Figure S2). The abbreviation NVA indicates the presence of nonivamide in the samples, while bnPEG-4 points to the presence of benzyl 1-(polyethylene glycol)-4. The solutions were kept at 4 °C for 24 h to obtain a transparent solution. All hydrogels were stored in the fridge until taken out for analysis.

**Table 1:** List of the Prepared Formulations

	Composition [parts (w/w)]			
Name	Pluronic F127	PPG-8	Nonivamide or bnPEG-4	Water
F127^a^	25	-	-	100
PPG5^a^	25	5	-	100
PPG10^a^	25	10	-	100
PPG15^a^	25	15	-	100
PPG16	25	16	-	100
PPG17	25	17	-	100
PPG18	25	18	-	100
PPG19	25	19	-	100
PPG20	25	20	-	100
NVA^a^	25	-	0.5	100
NVA PPG5^a^	25	5	0.5	100
NVA PPG10^a^	25	10	0.5	100
NVA PPG15^a^	25	15	0.5	100
NVA PPG16	25	16	0.5	100
NVA PPG17	25	17	0.5	100
NVA PPG18	25	18	0.5	100
NVA PPG19	25	19	0.5	100
NVA PPG20	25	20	0.5	100
bnPEG-4^b^	25	-	0.5	100
bnPEG-4 PPG5^b^	25	5	0.5	100
bnPEG-4 PPG10^b^	25	10	0.5	100
bnPEG-4 PPG15^b^	25	15	0.5	100

a Formulations were tested for *T*_gel_, release behavior, and rheological properties.

b Formulations were tested for *T*_gel_ and release behavior. The remaining formulations were only tested rheologically.

### 2.4 Determination of Gelation Temperature (*T*_gel_)

*T*_gel_ was determined by the tube inversion method, adapted from Abdeltawab et al.^7^ Samples (5 mL) of each formulation were placed in 20 mL vials and immersed in a water bath. The temperature was increased from 10 to 45 °C in 1 °C/min increments, with 5 min allowed for stabilization. *T*_gel_ was recorded when the sample did not flow upon tilting the vial. The measurements were performed in triplicate and are reported as mean ± SD in the results section.

### 2.5 Evaluation of Rheological Properties

#### 2.5.1 Determination of Viscosity

To determine the viscosity, 1 mL of the hydrogel at 4 °C was placed on the measuring plate set to 15 °C. The cone plate was lowered into the measuring position, and any excess sample was removed with a spatula. The solvent trap and side cap were installed. After a 3-min equilibration, the temperature-dependent viscosity of the formulations (η­(*T*)) was measured by gradually increasing the temperature from 15 to 40 °C in 1 °C/min increments, with a constant shear rate of γ = 10 s^–1^. The stable gel formation was defined as the plateau of the viscosity. The reference sample (neat Pluronic F127) was tested in triplicate to ensure reproducibility of the test (see Figure S3a).

#### 2.5.2 Determination of the Linear Viscoelastic Region (LVR)

To identify the linear viscoelastic range (LVR), 1 mL of the hydrogel at 4 °C was placed on the measuring plate set to 37 °C. The cone plate was lowered into the measuring position, and any excess sample was removed with a spatula. The solvent trap and side cap were installed. After a 3-min equilibration, shear strain was increased from γ = 0.01 to 100% at a fixed oscillation frequency of ω = 10 rad/s. The reference sample (neat Pluronic F127) was tested in triplicate to ensure reproducibility of the test (see Figure S3b).

#### 2.5.3 Determination of Storage Modulus (*G*’) and Loss Modulus (*G*″)

To determine the storage modulus (*G*’) and loss modulus (*G″*), 1 mL of the hydrogel at 4 °C was placed on the measuring plate set to 10 °C. The cone plate was then lowered into the measuring position, and any excess sample was removed with a spatula. The solvent trap and side cap were installed. After a 3-min equilibration, *G*’ and *G*″ values were measured from 10 to 40 °C with 0.5 °C/min increments at a fixed oscillation frequency of ω = 10 rad/s and shear strain set to 0.2%, as determined earlier by LVR. The sol–gel transition (*T*_sol–gel_) was determined as the crossover point between *G*’ and *G″*. *T*_gel_ was defined as the temperature where the *G*’ value reached a plateau. The reference sample (neat Pluronic F127) was tested in triplicate to ensure reproducibility of the test (see Figure S3c).

### 2.6 Release of Nonivamide and bnPEG-4 from Hydrogels

Release of nonivamide and bnPEG-4 was measured using the dialysis bag technique, with the protocol adapted from Giuliano et al.^5^ Before studying the release of bnPEG-4, the *T*_gel_ of the hydrogels was measured to ensure that the hydrogels were formed at or below 37 °C (Figure S4). Prior to the analysis, the linearity of the detector response was evaluated to establish sampling size, dilution, and injection volume (Figure S5). Dialysis bags were soaked in MilliQ water overnight and rinsed before use. An empty F127 hydrogel (control) and samples of each formulation (0.5 mL) were placed in a dialysis bag and secured tightly with knots and clamps to prevent leakage of the formulation and uncontrolled water penetration. The dialysis bags were transferred to a 15 mL Falcon tube and incubated at 37 °C overnight to ensure gelation. Prewarmed PBS buffer (10 mL buffer for control and bnPEG-4; 9.9 mL PBS and 100 μL of methanol for NVA) was added to the tube and incubated at 37 °C with constant stirring and agitation. Samples (10 μL) were taken at 0, 5, 15, 30, 45, 60, and 180 min. to analyze the release of nonivamide and bnPEG-4 into the PBS buffer. The aliquots were diluted with MilliQ water (1 mL) and analyzed by HPLC/MS. The release is reported as a percentage over time, with 100% release measured as the direct dissolution of 0.5 mL of a hydrogel with bnPEG-4 in 10 mL PBS and a hydrogel with nonivamide in 9.9 mL of PBS buffer and 100 μL of methanol. The PBS buffer samples were monitored by HPLC/MS for potential leakage of Pluronic F127. After the experiment, the samples were inspected for leakage and a decrease in volume. The measurements were performed in triplicates and reported as mean ± SD in the results section.

The partitioning coefficients (logP) of nonivamide and bnPEG-4 were predicted using ChemDraw (Revvity Signals Software, Inc.*).*

## 3 Results and Discussion

### 3.1 Effects of PPG-8 on *T*_gel_ and Viscosity

Hydrogels were prepared using the so-called “cold method”.^6^ Briefly, Pluronic F127 was dissolved in cold MilliQ water, and PPG-8 and nonivamide were added once the polymer dissolved. *T*_gel_ was determined using the tube inversion method (Figure 2a). Neat Pluronic F127 hydrogel showed a *T*_gel_ of 21.8 ± 0.2 °C (Figure 2b), consistent with previously reported values for unmodified Pluronic F127 hydrogels.^23^ Incorporating 5 parts (w/w) of PPG-8 (PPG5) raised *T*_gel_ to 22.3 ± 0.5 °C. A higher concentration of PPG-8, i.e., 10 parts (w/w) (PPG10), resulted in only a minor rise in *T*_gel_ to 22.6 ± 0.4 °C, while 15 parts (w/w) (PPG15) increased *T*_gel_ to 26.7 ± 0.5 °C.

**Figure 2:**
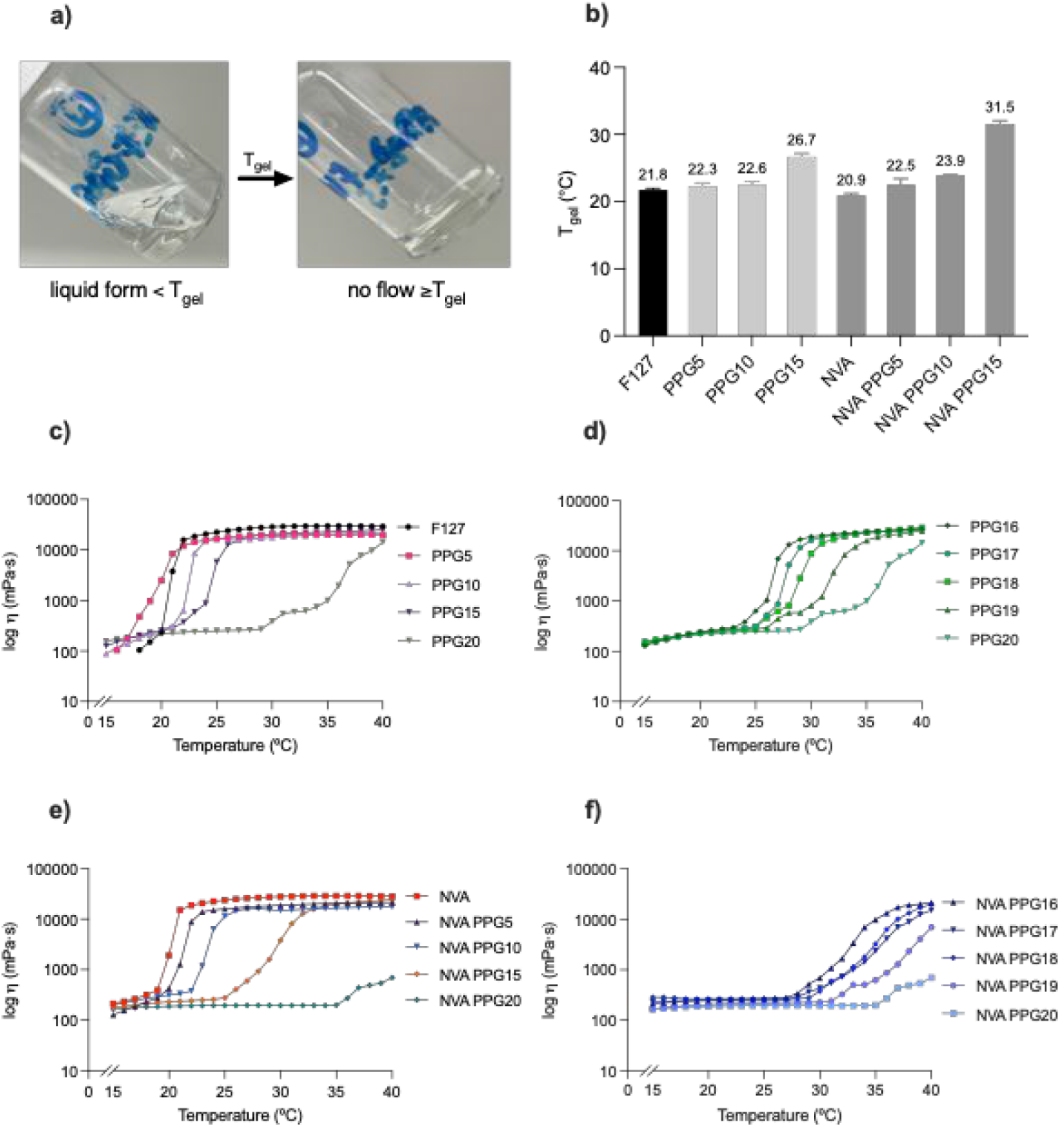
(a) Schematic representation of *T*_gel_ determination by the tube inversion method (Photo: Samol, Z.); (b) *T*_gel_ values for Pluronic F127, formulations containing 5, 10, and 15 parts (w/w) of PPG-8, and formulations with 0.5 parts (w/w) nonivamide and 5, 10, and 15 parts (w/w) PPG-8 (*n* = 3, data shown as mean value ± SD). Viscosity curves for (c) Pluronic F127 and formulations containing 5, 10, 15, and 20 parts (w/w) PPG-8; (d) 16 to 20 parts (w/w) PPG-8; (e) 0.5 parts (w/w) nonivamide and 5, 10, 15, and 20 parts (w/w) PPG-8; (f) 0.5 parts (w/w) nonivamide and 16 to 20 parts (w/w) PPG-8.

The introduction of small molecules to Pluronic hydrogels can lower *T*_gel_.^1^ This was observed for a hydrogel with just 0.5 parts (w/w) nonivamide (NVA), where *T*_gel_ decreased by nearly 1 °C to 20.9 ± 0.3 °C (Figure 2b). However, the presence of PPG-8 overrode this effect. With 5 parts (w/w) PPG-8, *T*_gel_ was 22.5 ± 0.9 °C (NVA PPG5), and with 10 parts (w/w) PPG-8, *T*_gel_ was 23.9 ± 0.1 °C (NVA PPG10). This could be attributed to the codissolution of nonivamide and PPG-8 in the micellar core.^18^ With 15 parts (w/w) of PPG-8, the dissolution was limited, resulting in *T*_gel_ reaching 31.5 ± 0.5 °C (NVA PPG15).

Interestingly, both samples with 20 parts (w/w) of PPG-8 (PPG20, NVA PPG20) did not form a gel at 45 °C when tested by the vial tilt method. This temperature cutoff is crucial, as the gel must form below body temperature for medicinal use. Therefore, we tested the hydrogels rheologically with increasing amounts of PPG-8 by 1 part (w/w), from 16 to 20 parts (w/w), to gain more insight into where *T*_gel_ remains below body temperature (Figure 2d–f). The plateau of the viscosity curve reflects stable gel formation and thus can provide an estimate of *T*_gel_.^24^ Consistent with the vial tilt method, the formulations with 20 parts (w/w) of PPG-8 (PPG20, NVA PPG20) showed no apparent plateau below 40 °C. For mixtures containing up to 19 parts (w/w) of PPG-8 (PPG16-PPG19), *T*_gel_ was below 37 °C (Figure 2d). For the formulations coloaded with nonivamide, even at 16 parts (w/w) of PPG-8 (NVA PPG16), no apparent viscosity plateau could be observed below 40 °C, indicating that no stable gel was formed (Figure 2f).

The presence of PPG-8 and nonivamide influenced the viscosity values. For instance, neat Pluronic F127 showed viscosities of around 44 mPa·s at 15 °C and 29760 mPa·s at 37 °C (Figure 2c). With 15 parts (w/w) of PPG-8 (PPG15), the viscosity increased to 125 mPa·s at 15 °C but decreased to 23399 mPa·s at 37 °C. Contrastingly, nonivamide alone increased the viscosity at both temperatures to 213 and 28723 mPa·s, respectively (Figure 2e). Similar observations were made by Djekic et al.^2^ when incorporating ibuprofen into Pluronic F127 hydrogels. However, PPG-8 limited this effect, and with 15 parts (w/w) of PPG-8 (NVA PPG15), the viscosity was lowered to 176 mPa·s at 15 °C and 21605 mPa·s at 37 °C.

The phase transition of Pluronic F127 from a solution (sol) to a semisolid (gel) depends on the CMC and temperature. Above the CMC and at low temperature, there is an equilibrium between Pluronic F127 unimers and spherical micelles formed due to the dehydration of the PPG blocks. With increasing temperature, the equilibrium shifts toward the formation of micelles and reducing the number of free unimers. The resulting increase in micelle volume fraction promotes the ordered packing of micelles into a crystal lattice, giving rise to a solid-like gel.^25,26^ Based on Russo et al.,^4^ any changes in solution composition can affect the CMC and consequently change *T*_gel_. In hydrogels containing only PPG-8 at concentrations up to 15 parts (w/w), *T*_gel_ was only slightly higher, as the intrinsic hydrophobicity of PPG facilitates its dissolution in the micellar core.^18^ With the coaddition of both PPG-8 and nonivamide, *T*_gel_ was significantly increased. The increase in *T*_gel_ for Pluronic F127 in the presence of additives like PEG and PPG has been reported earlier. The addition of PEG with MW = 400–4000 g/mol increased *T*_gel_, and additions above 20% PEG completely prevented gelation. This was attributed to volume exclusion and the disruption of micelle packing.^27^ Similar findings were reported by Lima et al.,^18^ who found that the incorporation of PPG400 (10–30%) into Pluronic F127 aqueous solutions (10–30%) lowered the CMC and increased T_gel_. It was also observed that the presence of PPG400 led to a transition in micelle packing from a face-centered cubic (fcc) structure to a less densely packed body-centered cubic (bcc) structure. Further studies are necessary to understand the variations in the microstructure of hydrogels upon the addition of PPG-8. Nevertheless, it is clear that the incorporation of PPG-8 over 15 parts (w/w) has a significant effect on *T*_gel_, allowing it to reach the functional range for most clinical applications, i.e., 25–37 °C.^6,7^

### 3.2 Determination of *G*′ and *Ǵ*″

The modulus tests were performed at 0.2% shear strain to ensure the internal structure of the gel was maintained during the measurements.^5,7^ This value of shear strain is far below the determined linear viscoelastic limit, i.e., 2% (Figure 3a) and is in line with other protocols for testing Pluronic F127.^4,24^ The low modulus values, where log *G*’ is below 10, corresponded to the liquid state of the sample (Figure 3b).^5,28^ The crossover point between *G*’ and ´*G*″ indicated the transition temperature, *T*_sol–gel_,^5^ where gelation began. The substantial jump in *G*’, followed by a plateau, reflected the formation of a solid-like gel at *T*_gel_.^5^

**Figure 3:**
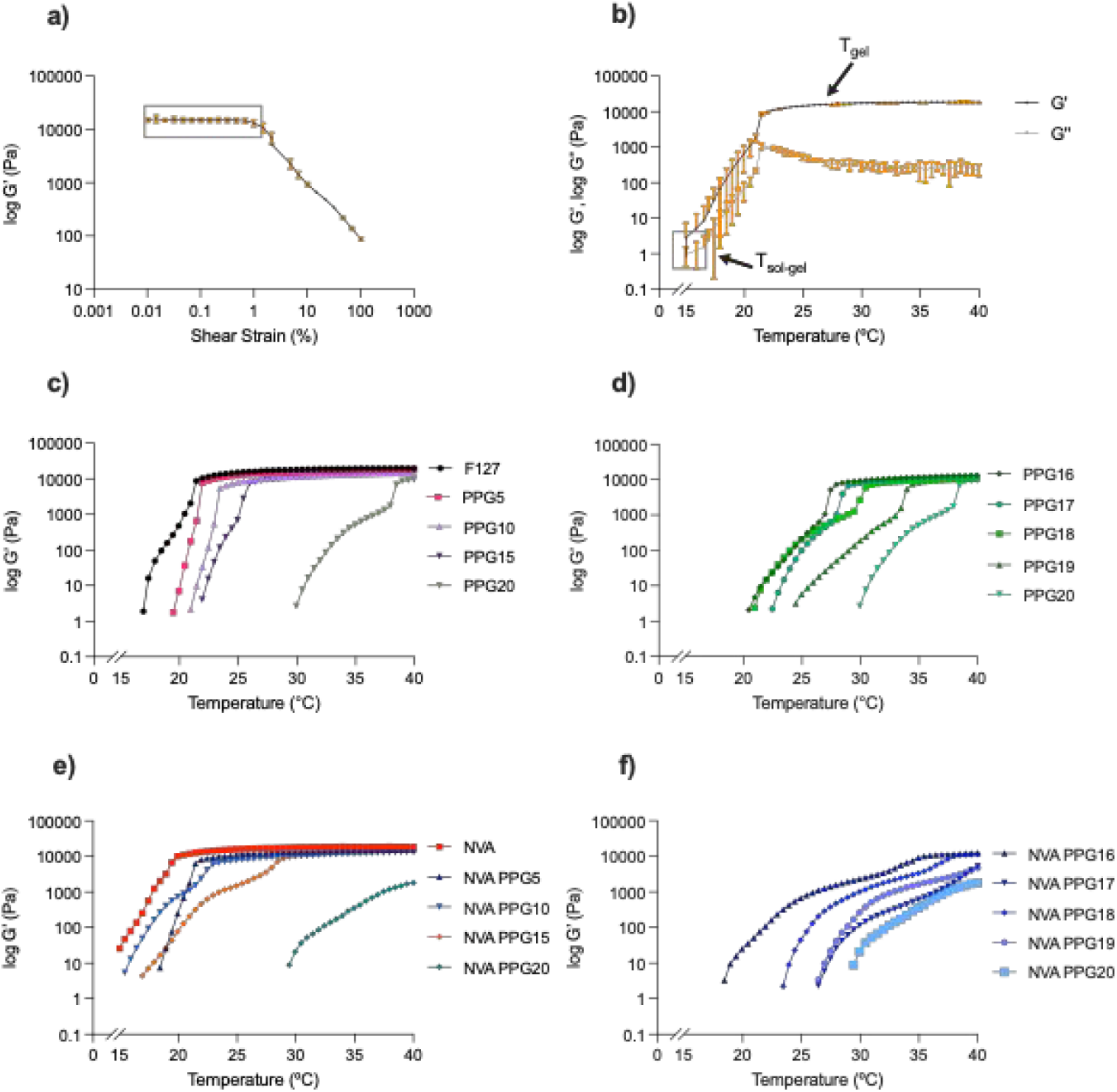
(a) The determination of the LVR region of neat Pluronic F127 (*n* = 3, data shown as mean value ± SD); (b) The determination of *T*_sol–gel_ and *T*_gel_ based on *G*’ and *G*″ curves. Neat Pluronic F127 *G*’ and *G*″ curves are shown as examples (*n* = 3, data shown as mean value ± SD). *G*’ and *G*″ of all samples see Figures S6 and S7; *G*’ curves for: (c) Pluronic F127 and formulations containing 5, 10, 15, and 20 parts (w/w) PPG-8; (d) 16 to 20 parts (w/w) PPG-8; (e) 0.5 parts (w/w) nonivamide and 5, 10, 15, and 20 parts (w/w) PPG-8; (f) 0.5 parts (w/w) nonivamide and 16 to 20 parts (w/w) PPG-8.

Increasing PPG-8 content raised *T*_sol–gel_ (Figure S6). Unmodified Pluronic F127 exhibited a *T*_sol–gel_ of around 15 °C, which increased to approximately 28 °C with 20 parts (w/w) of PPG-8 (PPG20). The addition of nonivamide (Figure S7) lowered *T*_sol–gel_ to approximately 12 °C (NVA), but with 20 parts (w/w) PPG-8 (NVA PPG20), it increased to around 30 °C. The crossover point and a rising *G*’ were observed for all tested samples, suggesting micellization and self-assembly.^5^

Contrastingly, not all samples reached a clear *G*’ plateau, meaning some samples did not form a stable gel network in the tested temperature range.^29^ No plateau was observed for the sample with 20 parts (w/w) PPG-8 (PPG20) (Figure 3c), consistent with the vial tilt method and viscosity results, which showed no gel formation at 45 °C. Samples with 5 to 19 parts (w/w) PPG-8 (Figure 3c,d) exhibited a plateau below 37 °C (PPG5, PPG10, PPG15, PPG16, PPG17, PPG18, PPG19), which is in line with the obtained viscosity curves (Figure 2c,d). The samples with nonivamide and up to 15 parts (w/w) PPG-8 (NVA PPG5, NVA PPG10, NVA PPG15) showed a *G*’ plateau (Figure 3e), consistent with the viscosity plateau (Figure 2e). For samples with 16 and 18 parts (w/w) PPG-8 (NVA PPG16, NVA PPG18), an onset of plateauing was observed (Figure 3f), suggesting the beginning of gelation at around 35 °C. This is in agreement with the rapid change in viscosity for samples with up to 19 parts PPG-8 (NVA PPG16, NVA PPG17, NVA PPG18, NVA PPG19) (Figure 2f) also indicating the start of gelation.

The maximum values of *G*’ (*G*’_max_) were used to estimate the stiffness of hydrogels.^5^ The neat Pluronic F127 hydrogel had a *G*’_max_ of around 19 kPa. With increasing PPG-8 content, *G*’_max_ decreased from 18 kPa at 5 parts (w/w) PPG-8 (PPG5) to 15 kPa at 10 parts (w/w) PPG-8 (PPG10), and to 13 kPa at 15 parts (w/w) PPG-8 (PPG15). Between 16 and 20 parts PPG-8 (w/w) (PPG16, PPG20), a further decrease in *G*’_max_ occurred from approximately 13 to 9 kPa. The same trend of decreasing *G*’_max_ was observed for samples that contained both nonivamide and PPG-8. *G*’_max_ decreased from 16 kPa at 5 parts (w/w) PPG-8 (NVA PPG5) to 13 kPa at 15 parts (w/w) PPG-8 (NVA PPG15). The presence of nonivamide alone had minimal impact *G*’_max_, as both the neat Pluronic F127 and the nonivamide-loaded sample (NVA) showed a *G*’_max_ of around 19 kPa. Similar results have been reported for other Pluronic F127 hydrogels containing small hydrophobic molecules.^28^

### 3.3 Release of Nonivamide and bnPEG-4 from Hydrogels

Pluronic F127 hydrogels can be formulated to deliver both hydrophilic and hydrophobic molecules. Hydrophilic molecules are solubilized in the aqueous solution between the hydrophilic PEG corona of the Pluronic micelles,^30^ while hydrophobic molecules undergo dissolution in the hydrophobic PPG core of the micelles.^1,31^ Despite their capacity to solubilize both hydrophilic and hydrophobic compounds, Pluronic hydrogels exhibit certain limitations that hinder optimal drug delivery and release. The release of drugs from Pluronic hydrogels is predominantly driven by gel dissolution in the surrounding medium and, to a much lesser extent, by gradual drug diffusion from the gel into the medium.^25,32^ Highly hydrophilic drugs, such as bupivacaine hydrochloride,^7^ suffer from a high initial burst release from the hydrogel once placed in an aqueous solution, while hydrophobic drugs are released very slowly into the water environment.^15^ Therefore, the use of additives can help tailor release rates.^33^

In this work, the release of two model compounds from Pluronic F127 hydrogels into PBS buffer was studied, namely nonivamide (Figure 4a) and bnPEG-4 (Figure 4c). The chosen compounds have comparable molecular weights (293.41 g/mol vs. 240.30 g/mol) but differ in the degree of hydrophobicity. The benzyl ring, together with the alkyl chain of nonivamide, leads to higher hydrophobicity compared to the benzyl ring combined with the ether chain of bnPEG-4. The difference in hydrophobicity can be reflected by the predicted partitioning coefficient values (logP). LogP for nonivamide equals 3.7, whereas the logP of bnPEG-4 is around 1. This approach allowed for the comparison of the influence of PPG-8 on the release of molecules with differing hydrophobicity.

**Figure 4:**
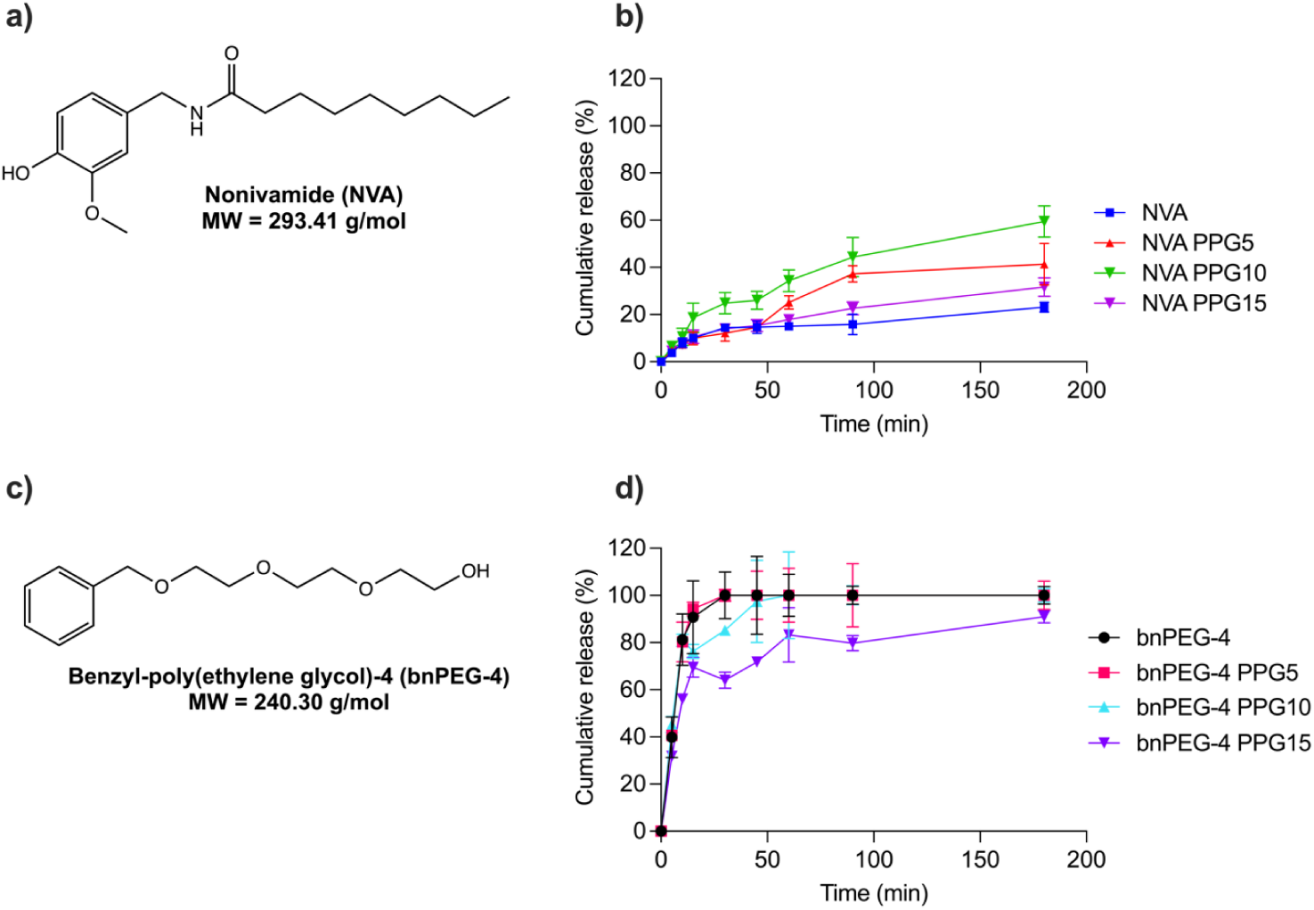
(a) Structure and molecular weight of nonivamide; (b) release of nonivamide from Pluronic F127 hydrogels containing 0 (blue), 5 (red), 10 (green), and 15 (purple) parts (w/w) PPG-8 (*n* = 3, data shown as mean value ± SD); (c) structure and molecular weight of bnPEG-4; (d) release of bnPEG-4 from Pluronic F127 hydrogels containing 0 (black), 5 (magenta), 10 (blue), and 15 (purple) parts (w/w) PPG-8 (*n* = 3, data shown as mean value ± SD).

Only about 20% of nonivamide was released into the PBS buffer over a 3 h interval from an unmodified Pluronic F127 (Figure 4b). Comparable results were reported for Pluronic hydrogels containing curcumin^15^ and rutin,^5^ which had a maximum release rates of 20–30% and 40–50%, respectively. This low release could be explained by the high viscosity of the unmodified F127 hydrogel (NVA) prepared at 25% parts (w/w) concentration (Figure 2e). The high initial viscosity was shown to impede the diffusion of drugs, which is expressed by their diffusion coefficients.^32,34^ Additionally, the preferential distribution of the hydrophobic nonivamide into the PPG micelle cores, rather than the hydrophilic phase of the gel, may also impact its release into the surrounding medium.^32^ PPG-8 significantly influenced the release of nonivamide. With the lowest addition of PPG-8 (NVA PPG5), the release was nearly doubled within the same time frame. When 10 parts (w/w) of PPG-8 (NVA PPG10) were added, the release further increased to about 60%. Similar effects have been observed with the addition of up to 10% PEG400, which was used to improve the solubility of hydrophobic curcumin^15^ and emodin.^23^ Ricci et al.^32^ described that the addition of 5% PEG400 to 25% F127 hydrogels loaded with lidocaine decreased the viscosity of the hydrogels. This consequently increased the diffusion coefficient of lidocaine from 1.64 × 10^–6^ cm^2^·s^–1^ for unmodified F127 to 2.70 × 10^–6^ cm^2^·s^–1^. However, further increasing the PPG-8 concentration to 15 parts (w/w) (NVA PPG15) resulted in only a slight increase in release, up to 30%, compared to the unmodified Pluronic F127, which released about 20% of the drug. A nearly 3-fold decrease in the release of nonivamide between 10 parts (w/w) and 15 parts (w/w) of PPG-8 (NVA PPG10, NVA PPG15) indicates possible phase separation between PPG-8 and Pluronic F127.

The phase separation of PPG-8 from the aqueous solution of Pluronic F127 below *T*_gel_ could diminish its positive effect on the viscosity of PPG-8. The viscosity between 35 and 40 °C of hydrogel with 15 parts (w/w) of PPG-8 (PPG15 NVA) was slightly higher than that with 5 and 10 parts (w/w) PPG-8, approaching that of neat F127 with increasing temperature (Figure 2e; no logarithmic scale shown in Figure S8b). Malmsten et al.^17^ found evidence of phase separation in aqueous solutions of Pluronic F127 with PPG4000 already at 1 wt %, no phase separation was observed for PPG400. However, the tested concentrations did not exceed 10 wt %.

Rheological studies can be applied to detect phase separation.^35^ The *G*’ curve of NVA PPG15 (Figure 3e) shows variation compared to hydrogels with up to 10 parts (w/w) PPG-8 (NVA PPG5, NVA PPG10). There is a clear indentation in the curve between 20 and 30 °C, indicating phase separation, which is not as pronounced at lower concentrations of PPG-8. The solubility of PPG in water decreases with increasing temperature and concentration.^36^ At 15 parts (w/w) PPG-8 (NVA PPG15), the concentration of PPG-8 might be high enough to lead to the separation of a PPG-8-rich phase from the aqueous solution of Pluronic F127. The observed decrease in G’_max_ might also point to phase separation, as the inclusion of a separate PPG-8-rich phase in the hydrogel matrix might weaken the hydrogel network. Similar trends in viscosity and *G*’ values were observed for hydrogels without nonivamide (Figure 2a; no logarithmic scale shown in Figure S8a). The viscosity of hydrogel with 15 parts (w/w) of PPG-8 (PPG15) was also higher than that of hydrogels with 5 and 10 parts (w/w) of PPG-8 (PPG5, PPG10). A clear change in the *G*’ curve was also noted for a higher concentration of PPG-8 at 20 parts (w/w) (PPG20). In this case, the presence of nonivamide might have led to the phasing out of PPG-8 already at a concentration of 15 parts (w/w) PPG-8.

In contrast, the more hydrophilic bnPEG-4 was almost completely released from unmodified Pluronic F127 (Figure 4d) within 30 min. The addition of 5 parts (w/w) of PPG-8 (bnPEG-4 PPG5) did not result in a significant change in the release. With the incorporation of 10 parts (w/w) of PPG-8 (bnPEG-4 PPG10), the initial burst release decreased to 60% within the first 10 min compared to over 90% for unmodified F127, followed by approximately 70% of bnPEG-4 being released within the first 20 min. Finally, the highest addition of 15 parts (w/w) (bnPEG-4 PPG15) reduced the burst release from 90% to 70% and achieved sustained release over 3 h, reaching up to 90%.

The effect of PPG-8 on the release strongly depends on the hydrophobicity of the loaded compound.^8,30^ It has been demonstrated previously that drug release from Pluronic hydrogels depends on the degree of hydrophobicity of the drug.^30^ The hydrophilicity of the drug dictates its location within the hydrogel matrix. With higher hydrophilicity, the drug will partition into the hydrophilic PEG corona rather than the PPG micelle core, leading to rapid release upon gel erosion. This mechanism explains the almost complete release of bnPEG-4 within 10 min. The addition of PPG-8 had a limited effect on bnPEG-4 release, only slightly reducing the burst release with 15 parts (w/w) PPG-8 (bnPEG-4 PPG15). Possibly, the presence of a separate PPG-8 phase might have locally limited gel dissolution and slightly hindered the release into the PBS buffer. In the case of nonivamide, the presence of PPG-8 led to lower viscosity and possibly less dense micelle packing,^18^ which might favor faster gel dissolution and, therefore, increase the release of nonivamide.^32^ The impact of PPG-8 is largely dependent on the hydrophobicity of the released molecule and its affinity toward the PEG corona or PPG core.

## 4 Conclusions

This work focused on the preparation and characterization of Pluronic F127 hydrogels modified with monodisperse PPG-8. *T*_gel_ and rheological properties of the prepared formulations were studied. The influence of the incorporation of a defined oligomer on the release of two model compounds, similar in molecular weight but differing in hydrophobicity, was also investigated, namely nonivamide and bnPEG-4.

To preserve optimal mechanical properties and viscosity, it is recommended to limit the addition to between 10 and 15 parts (w/w) of PPG-8 with 25 parts (w/w) of Pluronic F127. Introducing PPG-8 into nonivamide-loaded hydrogels increased the cumulative release from 20% to 60% and *T*_gel_ by 3 °C. The release studies showed that the effect of PPG-8 is largely dependent on the type of model compound being tested. PPG-8 can significantly increase the release of strongly lipophilic molecules but only slightly limit the initial burst release of more hydrophilic compounds. Further work is needed to fully understand the effect of PPG-8 on micelle formation and gelation at the microscopic scale. Additionally, different oligomer lengths could be studied to further modulate the properties of Pluronic F127.

The strategy of simple physical incorporation of PPG-8 oligomers offers the opportunity to tailor the properties of hydrogels in a controlled and cost-effective manner. Such an approach results in greater control over the chemical composition of additives, as well as ensuring high quality and reproducibility of the final formulations.

## Supplementary Material



## Notes

HPLC analyses of PPG-400, PPG-8, nonivamide, and bnPEG-4; comparison of viscosity and log *G*’/log *G*″ of neat Pluronic F127 hydrogels prepared with water and with the addition of 0.01 parts (w/w) of methanol; viscosity curves of neat Pluronic F127 (*n* = 3); *T*_gel_ of bnPEG-4-loaded hydrogels; evaluation of HPLC-MS detector linearity response for nonivamide and bnPEG 4 samples; log *G*’ and log *G*″ curves of neat F127 and formulations containing PPG-8 and nonivamide; viscosity curves without logarithmic scale of hydrogels containing PPG-8 and nonivamide (PDF)
