# Supplementary material for: The Use of Monodisperse Poly(propylene glycol)‑8 as a Polymeric Additive: Effect on the Gelation Temperature and Rheological Properties of Pluronic Hydrogels
Source: ACS Omega. 2025 Jul 28;10(31):34485–93. doi: 10.1021/acsomega.5c02773 (PMC12355303; doi:10.1021/acsomega.5c02773)
Supplement: Supplementary file 1 [file ao5c02773_si_001.pdf]

Supporting Information

The use of monodisperse poly(propylene glycol)-8 as a polymeric additive: Effect on the gelation temperature and rheological properties of Pluronic hydrogels

Zuzanna Samol[1,2], Erik Agner[2], Magne O Sydnes*[1,3]

[1]Department of Chemistry, Bioscience, and Environmental Engineering, University of Stavanger, Stavanger 4036, Norway
[2]Polypure AS, Martin Linges vei 25, Fornebu 1364, Norway
[3]Department of Chemistry, University of Bergen, Bergen 5020, Norway

Prior to hydrogel preparation, the additives, i.e., PPG-8 (Figure S1a), nonivamide (Figure S1c), and bnPEG-4 (Figure S1d) were analyzed by HPLC-MS. Retention times and mass-to-charge ratios (*m/z*) are annotated on the respective chromatograms and spectra. Additionally, polydisperse commercial PPG400, used as the source for monodisperse PPG-8, was analyzed (Figure S1a).

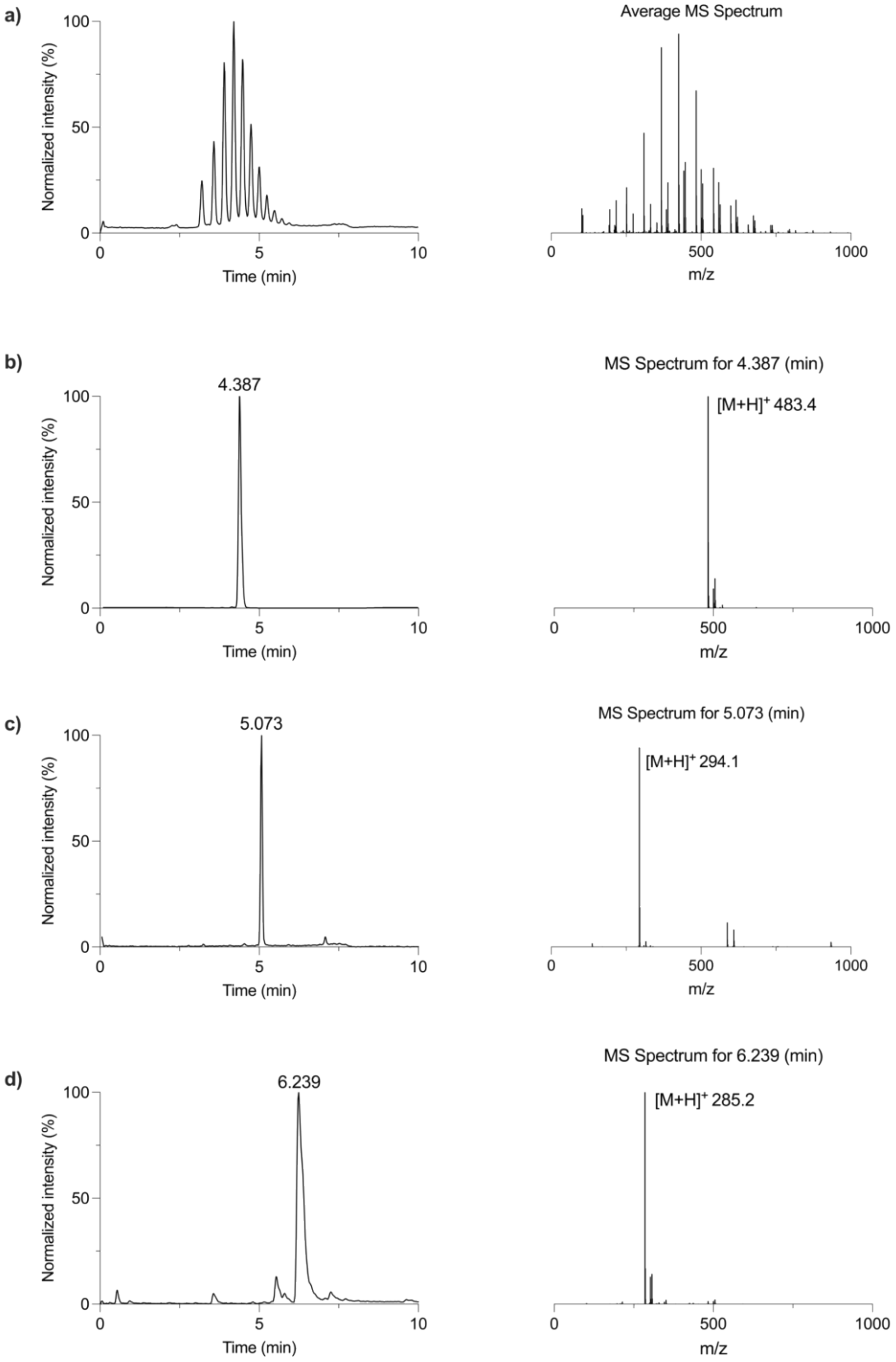

Figure S1. HPLC-MS analysis of PPG400 and hydrogel additives. Left chromatograms and right MS spectra. A) commercial PPG400; B) PPG-8; C) nonivamide; D) bnPEG-4.

To ensure solubilization of the hydrophobic nonivamide, the hydrogels containing nonivamide were prepared with 0.01 parts (w/w) addition of MeOH. Neat Pluronic F127 hydrogels prepared with water and water with 0.01 parts (w/w) MeOH (Figure S2) were tested rheologically to ensure no differences in gelation properties have occurred.

a)

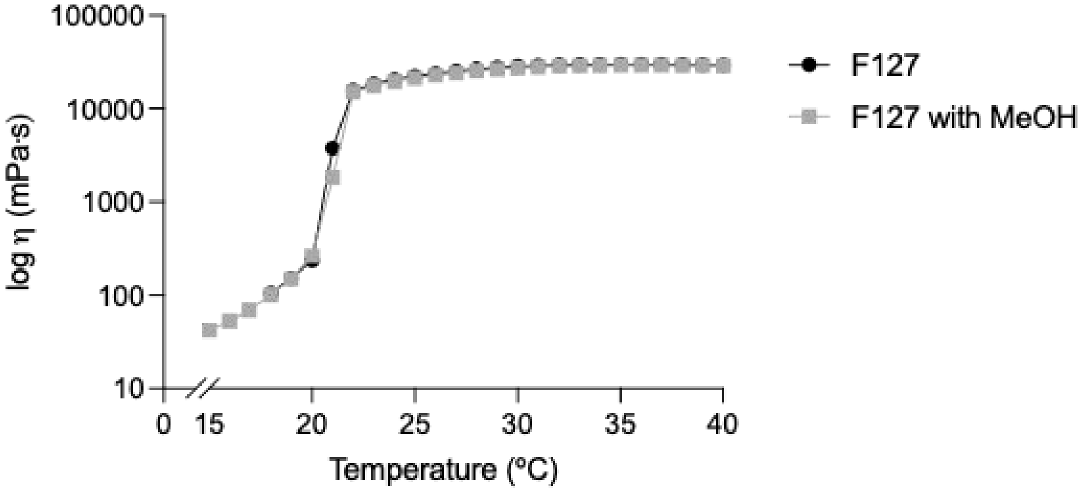

b)

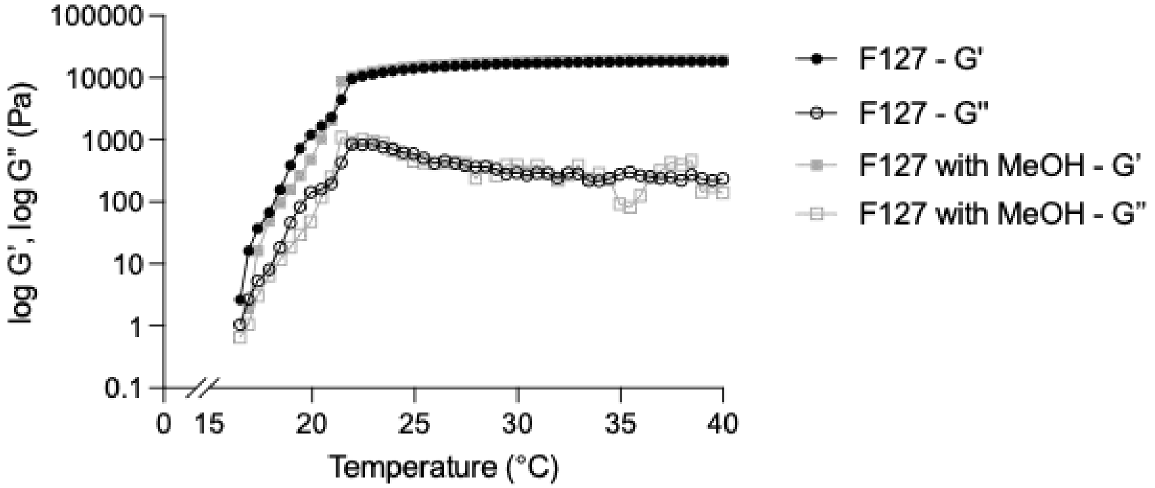

Figure S2. Comparison of Pluronic F127 hydrogels prepared with water vs. with water and addition of 0.01 parts (w/w) MeOH. A) Viscosity curves of neat F127 hydrogels prepared with water (black dots) and with addition of MeOH (grey squares). Error bars were removed for clarity. B) G' and G'' curves of F127 hydrogels prepared with water (black filled and empty dots) and with addition of MeOH (grey filled and empty squares). Error bars were removed for clarity.

The neat Pluronic F127 hydrogel (25 parts (w/w)) was tested in triplicate to ensure reproducibility of each rheological test (Figure S3).

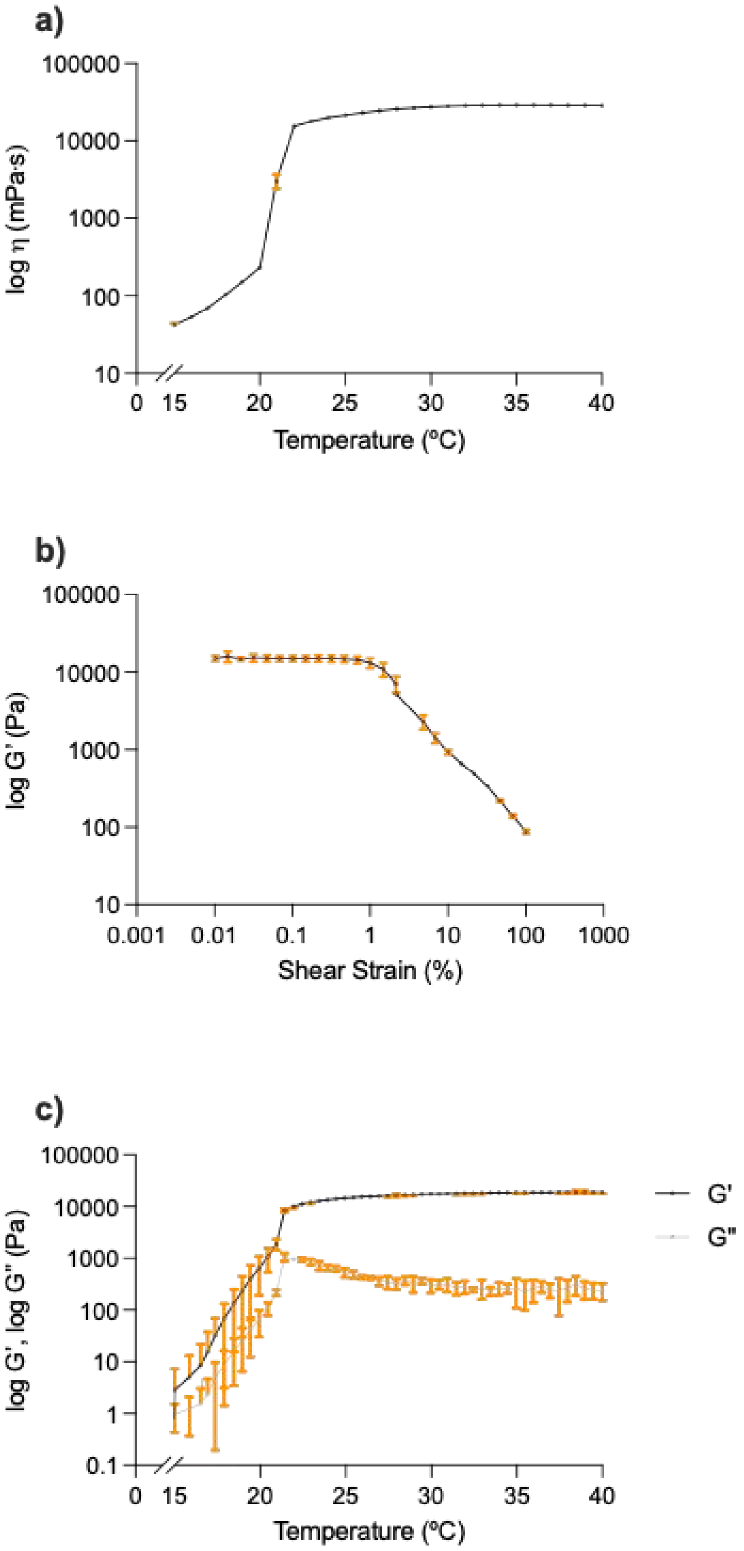

Figure S3. Evaluation of rheological testing protocols on neat Pluronic F127: A) Viscosity curves of neat Pluronic F127 (n = 3, data shown as mean value ± SD); B) Shear strain sweep used to determine linear viscoelastic limit of

neat Pluronic F127 (n = 3, data shown as mean value ± SD); C) G′ and G′′ curves of neat Pluronic F127 (n = 3, data shown as mean value ± SD.

Before the release studies, the T_{gel} of Pluronic F127 hydrogels containing bnPEG-4 was assessed by the vial tilt method (Figure S4) to ensure that the tested hydrogels were formed below 37 °C, i.e., the temperature at which the release was measured. Prior to studying the release of nonivamide and bnPEG-4 from the hydrogels, the evaluation of the HPLC-MS detector response for samples of nonivamide (Figure S5a) and bnPEG-4 (Figure S5b) was evaluated. This allowed to find suitable sampling size and dilution of the aliquots analyzed via HPLC-MS.

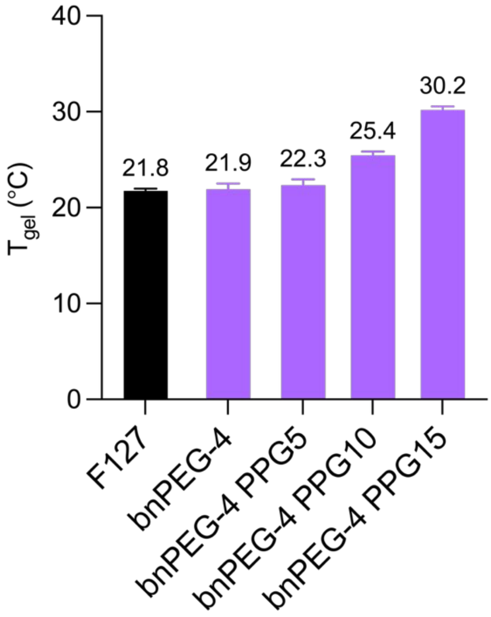

Figure S4. T_{gel} values for Pluronic F127, formulations with 0.5 parts (w/w) bnPEG-4 and 5, 10, and 15 parts (w/w) PPG-8 (n = 3, data shown as mean value ± SD

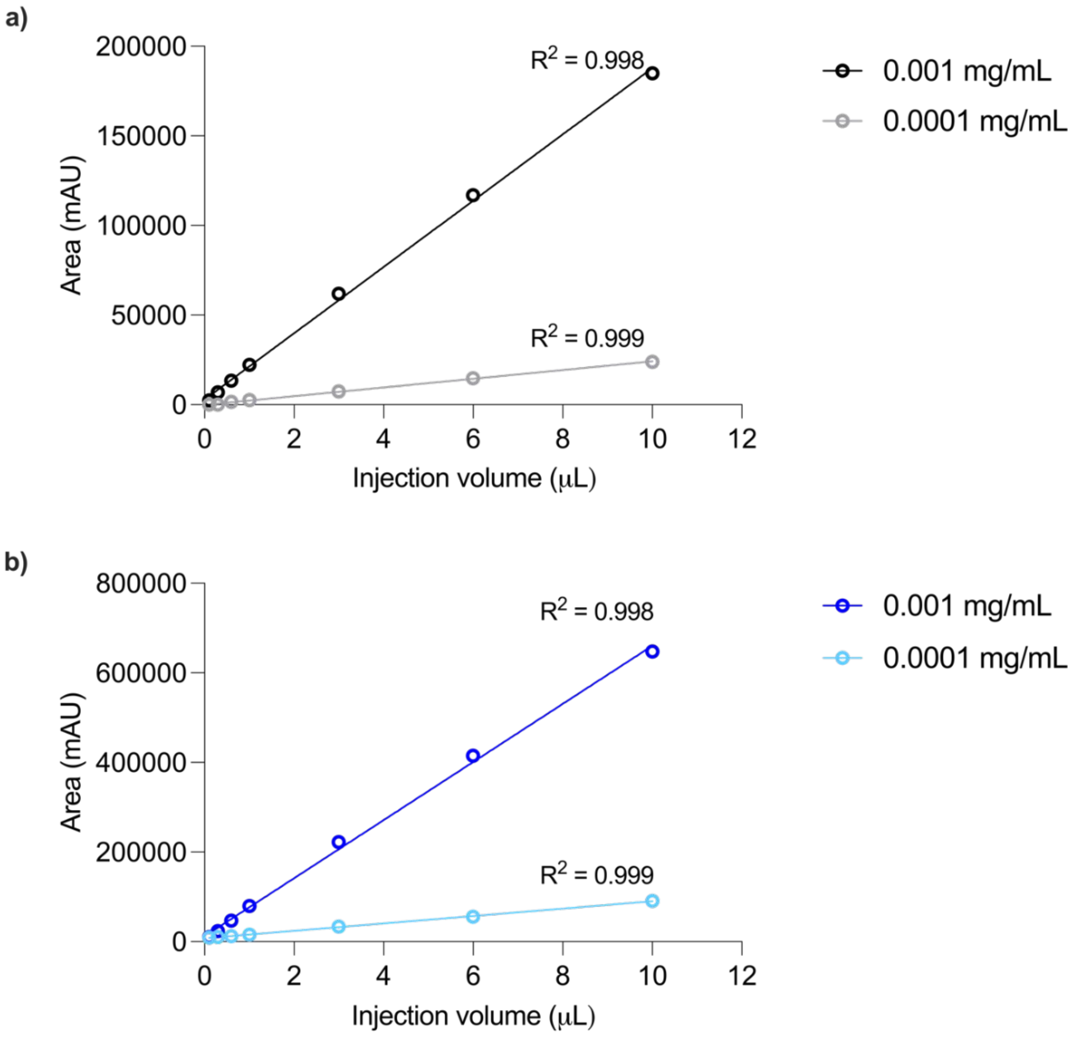

Figure S5. Evaluation of linearity response of the HPLC-MS detector for a) nonivamide; b) bnPEG-4.

The moduli measurements of Pluronic F127 hydrogels with PPG-8 are shown in Figure S6. The Pluronic F127 hydrogels loaded with PPG-8 and nonivamide are shown in Figure S7. The data points for log G' and G'' < 1 were removed for clarity due to meaningful variation in value at the liquid state of the samples.

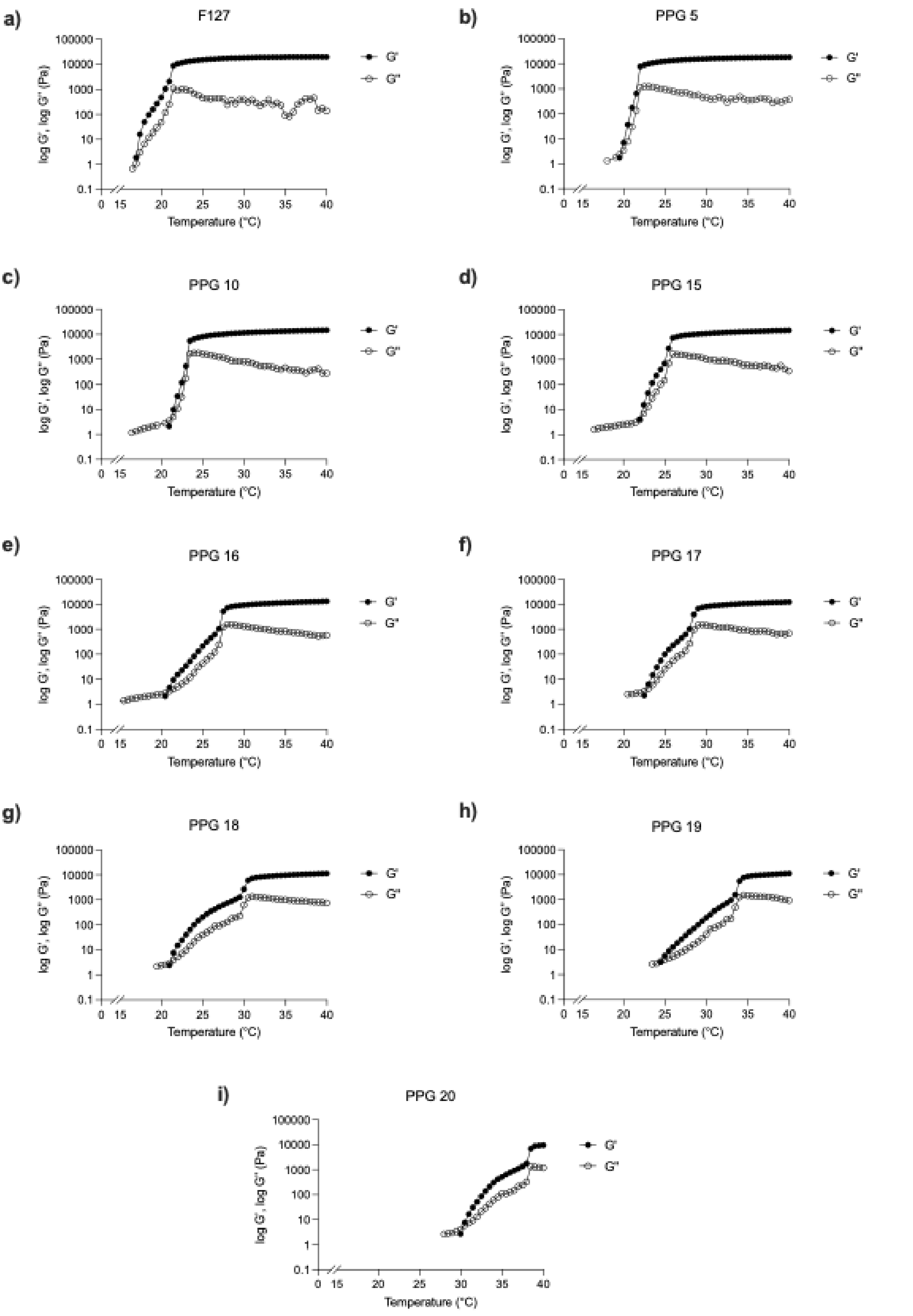

Figure S6. G' and G'' curves for Pluronic F127 hydrogels modified with PPG-8: a) Neat Pluronic F127 (error bars removed for clarity); b) 5 parts (w/w) PPG-8; c) 10 parts (w/w) PPG-8; d) 15 parts (w/w) PPG-8; e) 16 parts (w/w) PPG-8; f) 17 parts (w/w) PPG-8; g) 18 parts (w/w) PPG-8; h) 19 parts (w/w) PPG-8; i) 20 parts (w/w) PPG-8.

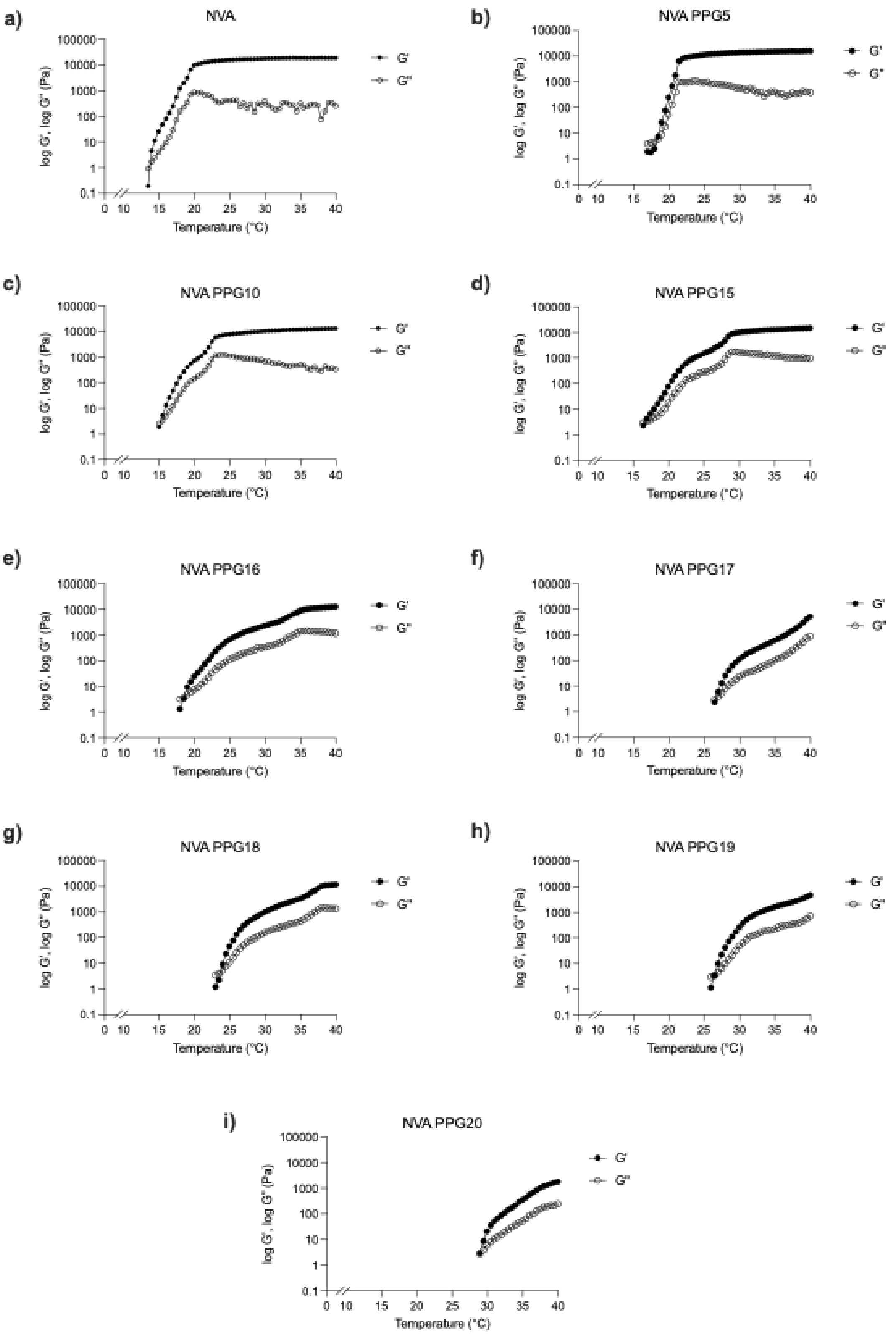

Figure S7. G' and G'' curves for F127 hydrogels with nonivamide and PPG-8: a) Neat F127 with nonivamide; b) synthetic nonivamide and 5 parts (w/w) PPG-8; c) nonivamide and 10 parts (w/w) PPG-8; d) nonivamide and 15 parts (w/w) PPG-8; e) nonivamide and 16 parts (w/w) PPG-8; f) nonivamide and 17 parts (w/w) PPG-8; g) nonivamide and 18 parts (w/w) PPG-8; h) nonivamide and 19 parts (w/w) PPG-8; i) nonivamide and 20 parts (w/w) PPG-8.

The viscosity measurements in function of temperature of Pluronic F127 hydrogels loaded with PPG-8 are shown in Figure S8a. The viscosity measurements of Pluronic F127 hydrogels co-loaded loaded with PPG-8 and nonivamide are shown in Figure S8b. The logarithmic scale was omitted to better visualize differences in viscosity between 30 and 40 °C.

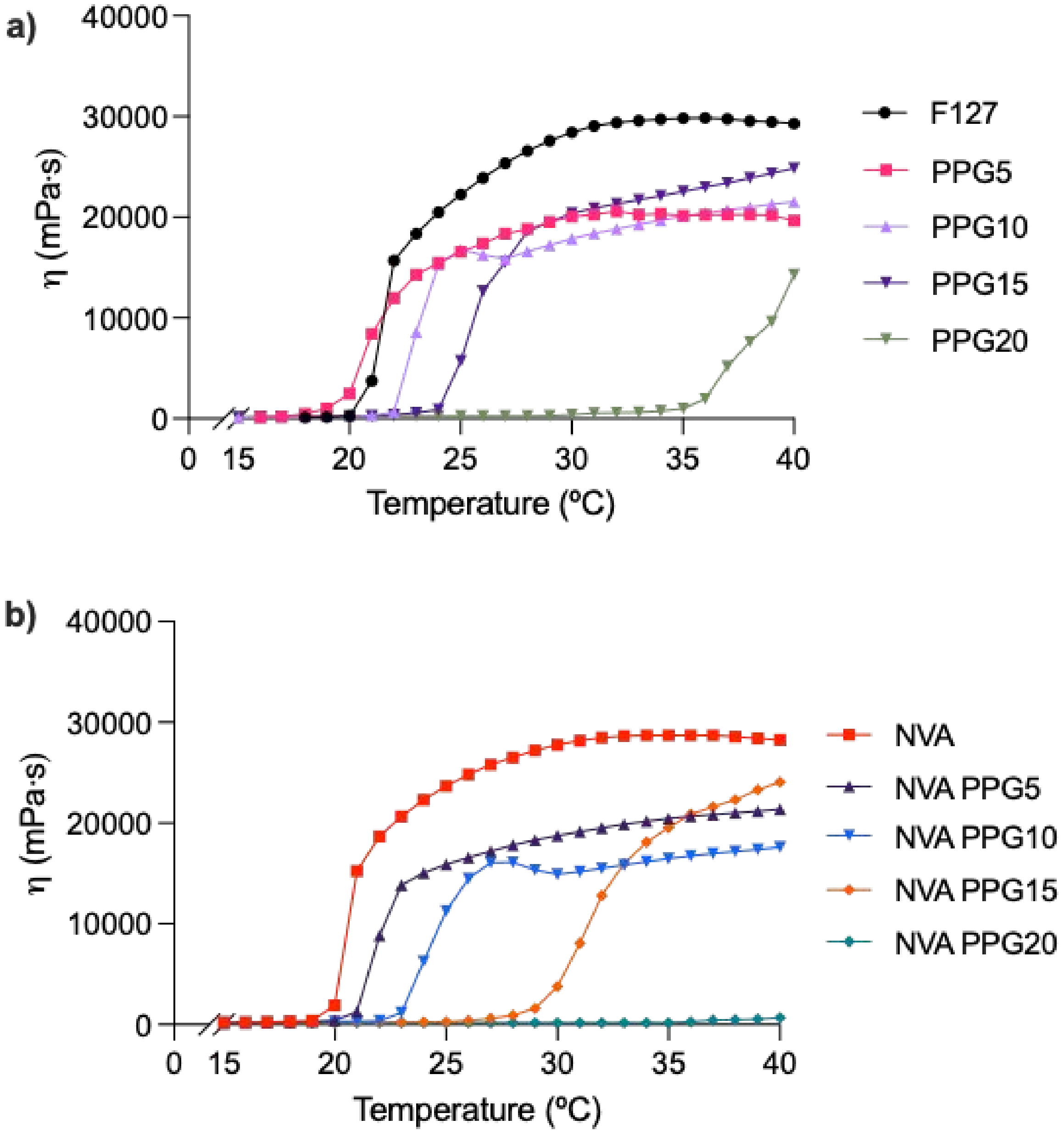

Figure S8. Viscosity curves for a) Pluronic F127 and formulations containing 5, 10, 15, and 20 parts (w/w) PPG-8; b) 0.5 parts (w/w) nonivamide and 5, 10, 15, and 20 parts (w/w) PPG-8; f) 0.5 parts (w/w) nonivamide and 16 to 20 parts (w/w) PPG-8.
